# Prevalence of anxiety, sleep bruxism and temporomandibular disorders during COVID-19 in Qatari children and adolescents: a cross-sectional study

**DOI:** 10.1007/s40368-023-00847-6

**Published:** 2023-10-17

**Authors:** H. Nazzal, M. Baccar, T. Ziad, T. Al-Musfir, B. Al Emadi, M. Matoug-Elwerfelli, S. Narasimhan, Y. Khan, S. Reagu

**Affiliations:** 1 Hamad Dental Centre, Hamad Medical Corporation, Doha, Qatar; 2 College of Dental Medicine, QU Health, Qatar University, Doha, Qatar; 3 Department of Psychiatry, Hamad Medical Corporation, Doha, Qatar; 4 NYU Langone Dental Medicine, Advanced Education in Pediatric Dentistry, Brooklyn, NY USA; 5 Weill Cornell Medicine, Doha, Qatar; 6 Pediatric Dental Services, Sun Life Health, Casa Grande, Arizona USA

**Keywords:** Anxiety, Bruxism, COVID-19, Dietary habit, Oral health, Paediatric dentistry, Temporomandibular disorders

## Abstract

**Purpose:**

Understanding the impact of coronavirus disease-2019 (COVID-19) pandemic social restrictions on the lives of children and adolescents is of utmost importance to enable timely diagnosis and treatment. Therefore, the aim of this study was to explore the prevalence of anxiety, sleep bruxism, temporomandibular disorders (TMD) and change in dietary and brushing habits and their association with COVID-19 social restrictions.

**Methods:**

Parents of fit and healthy Qatari children and adolescents were recruited and interviewed by the research team, whereby validated questioners were used to assess the prevalence of children’s/adolescents’ anxiety, sleep bruxism and TMD. Furthermore, changes in dietary and brushing habits were also evaluated.

**Results:**

A total of 199 parents of children and adolescents (mean age = 9.3 ± 3.2 years old) were included. Overall anxiety symptoms, sleep bruxism and TMD were evident in 29.6%, 5.7% and 23.1%, respectively. An increased consumption of food, sweets and worsening of brushing habits were evident in 51.8%, 62.8% and 31.2%, respectively.

**Conclusion:**

Within the limitations of this study, pandemic-related social restrictions could result in elevated levels of anxiety, specifically, social phobia, amongst children and adolescents, which could inevitably lead to unwanted dental consequences.

## Introduction

Anxiety disorders are one of the most common mental disorders with onset during childhood and a prevalence ranging between 10 and 30% (Kessler et al. 2012a, 2012b). Psychological stress, particularly when associated with symptoms of anxiety and depression, can play an important role as a predisposing, precipitating and perpetuating factor contributing to the development of multiple somatic symptoms including the ones related to specific dental disorders (Colonna et al. 2021). During the peak of coronavirus disease-2019 (COVID-19) pandemic, the State of Qatar, like most other countries, undertook unprecedented measures such as closure of all public facilities including schools, shopping malls and leisure centres to contain the spread of this highly contagious virus (Reagu et al. 2021). These measures resulted in almost complete home isolation of children and adolescents for approximately 6 months (March to September 2020). Consequently, online (distant) learning became the new norm for thousands of school-aged children and adolescents.

Social restrictions such as school closure and cancellation of outdoor activities, hence limited social interactions with friends, have been shown to be associated with severe distress, including anxiety symptoms and low mood in individual of all ages (Abdelrahman et al. 2021; Reagu et al. 2021). Although worrying is considered a normal and adaptive component of emotional development in children (Muris et al. 1998), excessive and/or persistent worrying can become pathological and interfere with the child’s functioning (Vasey and Daleiden 1994). Indeed, COVID-19 has significantly disrupted the usual routines of children and adolescents and have placed them in hitherto exceptional circumstances in which increase worrying could cause distress and mental health problems (Fegert et al. 2020). More specifically, the increase in anxiety disorders amongst children, due to the risk of infections and associated illness, is of clinical relevance (Khan et al. 2021).

Although evidence during the COVID-19 pandemic suggests a positive association of an increase in the frequency of temporomandibular disorders (TMD) symptoms and bruxism behaviour amongst adults with worsening psychosocial status (Colonna et al. 2021), data in children are deficient. In addition, evidence from a recent global scoping review based on self-reported data of 23 studies suggests a negative impact of COVID-19 pandemic on dietary habits of individuals (Bennett et al. 2021). The reduction in fresh produce and increase in comfort foods were associated with other unwanted lifestyle consequences such as limited physical activity and weight gain (Bennett et al. 2021). The hypothesis of this study was that the prevalence of anxiety, sleep bruxism and TMD can increase as a possible consequence to COVID-19 social restrictions in children and adolescents. Therefore, the primary aim of this study was to explore the prevalence rates of elevated symptoms of anxiety, sleep bruxism, TMD and any probable, relevant associations between them, likely associated with social restrictions during COVID-19 pandemic amongst a cohort of children and adolescents. The secondary aim was to explore the impact of COVID-19-related social restrictions on dietary and brushing habit changes amongst the same cohort.

## Materials and methods

### Ethical approval and study design

This cross-sectional study was performed in adherence to the ethical principles expressed in the revised Declaration of Helsinki (World Medical Association 2013). The research protocol was approved by the Medical Research Centre and the Institutional Review Board of Hamad Medical Corporation, Qatar (MRC-01–20-923).

### Inclusion criteria

Parents of fit and healthy children/adolescents between the age of 6 and 16 years with a good command of English and/or Arabic languages were invited to take part in this study. Children/adolescents with a history of established mental or behavioural disorders, sleep disturbances or TMD problems documented or confirmed by parents prior to the start of the COVID-19 social restrictions (March 2020) were excluded.

### Sample size calculation

The sample size was calculated using an online power and sample size calculator of prevalence studies (http://sampsize.sourceforge.net/iface/), with a 5% precision, a 15% prevalence of sleep bruxism (Fonseca et al. 2011)/ TMD (Wu and Hirsch 2010) and 95% confidence interval. The calculation resulted in an estimated sample size of n = 196 participants.

### Recruitment and data collection

Parents of children attending two paediatric dental centres at Hamad Dental Services (Hamad Dental Centre and Al-Khor hospital) were approached to participate in this study between January and September 2021. An informed written parental consent was obtained from all participants prior to enrolment in the study. Data collection was performed by two teams. The dental team recruited the parents and assessed the dental component of this project, whilst a psychiatric specialist contacted the parents and assessed children’s anxiety.

Prior to commencing the study, all members of the research team involved in data collection underwent a training and calibration session by the principal investigators (HN and SR) to ensure accurate and uniform data collection. During the training and calibration sessions, the principal investigators discussed each question in detail clarifying any ambiguity in question write up. Where necessary, trial interviews with patients (not included in the study), in the presence of the principle investigator, were conducted.

#### Dental component

This component was performed by two paediatric dental consultants, a paediatric dental specialist and an orthodontic consultant via face-to-face interviews when the restrictions eased resulting in phased return of dental services, in-person schoolings and other in-person facilities. During the second and third COVID-19 social restriction waves, recruitment and the dental component were performed via telephone interviews by the same dental team. The data collection tool was a composite instrument incorporating several aspects as follows:

- Demographic data: child’s age, gender, nationality, any pre-existing mental health conditions, and any history of a COVID-19 infection in the family.
- Development or change in sleep bruxism habits as described by Serra-Negra and colleagues (Serra-Negra et al. 2009, 2010). The diagnosis of sleep bruxism was in accordance with the American Academy of Sleep Medicine (AASM) classification criteria, with the severity of sleep bruxism defined in Table 1, with questions assessing children’s history of audible teeth grinding at night. In addition, questions pertaining to the family’s sleeping arrangements such as parent and child bedroom proximity (next door, same room, down the hall and different floor), parent and child bedroom door closure status (both doors closed, both doors open, either doors open) and the frequency of parental observation of their children during the night (once, twice, thrice, more than three times and do not check) were also included.
- Presence and severity of TMD utilising the Fonseca’s Anamnestic Index (FAI) questionnaire. The FAI is a validated self-rated questionnaire used for screening children/adolescents (Rigoldi Bonjardim et al. 2008; de Santis et al. 2014). The questionnaire consists of ten questions with “yes”, “no” or “sometimes” answers which are assigned values of “10”, “0”, “5”, respectively. The sum of the values is used to classify individuals into four categories: absence of TMD (0–15), mild TMD (20–45), moderate TMD (50–65) and severe TMD (70–100).
- Child’s dietary and brushing habits: questions assessing change in children’s oral hygiene and dietary habits during the COVID-19 social restrictions were developed and piloted on a group of parents (not included in the study). The questions included: did your child start eating more food during the COVID-19 pandemic restrictions, did your child start eating more sweets during the COVID-19 pandemic restrictions, did your child start drinking more carbonated drinks during the COVID-19 pandemic restrictions, and did your child’s brushing habit worsen during the COVID-19 pandemic restrictions. The answers involved a five-point Likert scale of strongly agree, agree, neither agree or disagree, disagree and strongly disagree.

**Table 1:** The classification and definition of the severity of sleep bruxism according to the American Academy of Sleep Medicine (AASM)

Severity	Definition
Mild	Episodes occur less than nightly, without evidence of impairment of psychosocial functioning
Moderate	Episodes occur nightly, with evidence of mild impairment of psychosocial functioning
Severe	Episodes occur nightly, with evidence of TMJ disorders, other physical injury, or moderate or severe impairment of psychosocial functioning

#### Anxiety disorder level component

This component was performed by a psychiatric specialist via telephone interview throughout the study period. The interview focussed on assessing children/adolescents for elevated symptoms of anxiety using the original English and validated Arabic version of the Spence Children’s Anxiety Scale-parent version (SCAS-P) (Nauta et al. 2004; Thabet et al. 2006). The SCAS was developed to assess the severity of anxiety symptoms broadly in line with the dimensions of anxiety disorder proposed by the Diagnostic and Statistical Manual of Mental Disorders (DSM)-IV (Spence 1997, 1998). This scale has been recently used in assessing children’s elevated anxiety symptoms during COVID-19 pandemic (Khan et al. 2021). The scale assesses 6 anxiety domains listed in Table 2, using a total of 38 items randomly allocated within the questionnaire assessing; panic attack and agoraphobia (9 items), separation anxiety disorder (6 items), obsessive compulsive problems (6 items), social phobia (6 items), generalised anxiety disorder (6 items) and physical injury fears (5 items). Parents are asked to rate the frequency of each symptom on a four-point scale including never (0), sometimes (1), often (2) and always (3). The cut-off points for elevated levels of anxiety are established using age range (younger age (≤ 9) and older age (≥ 10) groups) and gender-specific T-scores. The T-scores were developed, within the index, to establish whether anxiety symptoms are elevated above what would be regarded as normal levels in equivalent age and gender groups within the community (Khan et al. 2021). Raw total scores for overall anxiety and specific anxiety disorders obtained after adding responses on the scale are considered elevated if their equivalent T-scores are above 60.

**Table 2:** Anxiety disorder domains and definitions

Definition	
Generalised anxiety disorder^*^	Generalised and persistent anxiety but not restricted to, or strongly predominating in, any specific circumstances
Panic/agoraphobia^*^	Panic: recurrent attacks of severe anxiety Agoraphobia: a well-defined cluster of phobias embracing various fears such as leaving home, entering shops, public places and crowds, or travelling alone via buses, trains or planes
Social phobia^*^	Fear of scrutiny by other individuals resulting in avoidance of social situations
Separation anxiety disorder^*^	Fear of separation constitutes the main focus of the anxiety
Obsessive compulsive disorder^*^	The essential feature is recurrent obsessional thoughts or compulsive acts
Physical injury fears	A type of specific phobia in which the fear is driven by irrational worrying about experiencing physical harm or injury

^*^Definitions based on International Statistical Classification of Diseases and Related Health problems 10^th^ revision (World Health Organisation 2015)

### Statistical analysis

Data analysis was performed using the Statistical Package for the Social Sciences (SPSS; version 25 IBM Inc., Chicago, USA). Descriptive statistics are presented as mean and standard deviation (SD) or frequencies and percentages, depending on distribution of data. Logistic (binomial) regression analysis models were conducted to assess an association between various independent variables upon a dependent variable of interest such as anxiety symptoms, bruxism and TMD status. Answers to change of dietary and brushing habits were grouped into binary results where those who answered strongly agree and agree were combined into non-favourable changes in habits and those who chose neither agree or disagree, disagree or strongly disagree were grouped into favourable changes in habits. Age was considered as continuous variable and the reference category used for all the categorical variables (anxiety status and its sub-domains) were absence of the respective conditions. A two-tailed *p* value ≤ 0.05 was considered as statistically significant.

## Results

A total of 199 participants were included with a mean age of 9.3 ± 3.2 years old, detailed descriptive data are summarised in Table 3. Overall anxiety symptoms were evident in approximately a third of participants (*n* = 59, 29.6%). Anxiety domains such as physical injury fears, social phobia and separation anxiety disorders were most reported (Table 3). On comparison between the two age groups, elevated symptoms of overall anxiety disorders were reported in the younger age (≤ 9) group (*n* = 44, 36.9%) compared to the older age (≥ 10) group (*n* = 15, 19%) (Table 4).

**Table 3:** Participants’ demographics

		Frequency (Percentage)
Age category^#^	Age ≤ 9	119 (60.1%)
	Age ≥ 10	79 (39.9%)
Child’s gender^#^	Female	87 (44.2%)
	Male	110 (55.8%)
Child’s nationality^#^	Non-Qatari (expat)	48 (24.2%)
	Qatari	150 (75.8%)
History of a COVID-19 infection in the family	No	122 (61.3%)
	Yes	77 (38.7%)
Bruxism status^#^	No	183 (94.3%)
	Yes	11 (5.7%)
Temporomandibular disorders status	No	153 (76.9%)
	Yes	46 (23.1%)
Overall anxiety status	Absent	140 (70.4%)
	Present	59 (29.6%)
Panic/agoraphobia status	Absent	154 (77.4%)
	Present	45 (22.6%)
Separation anxiety disorder status	Absent	129 (64.8%)
	Present	70 (35.2%)
Physical injury fear status	Absent	124 (62.3%)
	Present	75 (37.7%)
Social phobia status	Absent	127 (63.8%)
	Present	72 (36.2%)
Obsessive compulsive disorder status	Absent	156 (78.4%)
	Present	43 (21.6%)
Generalised anxiety disorder status	Absent	133 (66.8%)
	Present	66 (33.1%)

^**#**^Counts do not sum to 199 in some categorical variables due to missing data

**Table 4:** Frequency of number of children, per age category, demonstrating symptoms of anxiety disorders

Anxiety domains	Age ≤ 9, *n* = 119	Age ≥ 10, *n* = 79	Total, *n* = 199
	Frequency^#^ (percentage)	Frequency^#^ (percentage)	Frequency^#^ (percentage)
Overall anxiety disorder	44 (36.9%)	15 (19%)	59 (29.6%)
Panic/agoraphobia	29 (24.3%)	16 (20.3%)	45 (22.6%)
Separation anxiety disorder	51 (42.8%)	19 (24.1%)	70 (35.1%)
Physical injury fears	57 (47.9%)	18 (22.8%)	75 (37.6%)
Social phobia	41 (34.4%)	31 (39.2%)	72 (36.1%)
Obsessive compulsive disorder	23 (19.3%)	20 (25.3%)	43 (21.6%)
Generalised anxiety disorder	49 (41.1%)	17 (21.5%)	66 (33.17%)

^**#**^The frequency denotes the counts (percentages) of children in each age category (≤ 9, ≥ 10, or total) who have the respective anxiety domain

According to the AASM criteria, signs of sleep bruxism were manifested in only 5.7% (*n* = 11) of the included participants with a majority of mild severity. Due to the low number of participants with a sleep bruxism outcome in this study, further logistic regression analysis of possible associated factors was not performed. Parental and child bedroom arrangements showed that the majority reported room proximity of either next door (*n* = 87, 56.86%) or down the hall (*n* = 41, 26.80%). The reminder reported either same room (*n* = 21, 13.73%) or on a different floor level (*n* = 4, 2.61%). In terms of parent and child bedroom door status, keeping both doors open (*n* = 79, 53.02%) was reported by approximately half of the parents. The remaining half of parents reported having both doors closed (*n* = 43, 28.86%) and one door open (*n* = 27, 18.12%). A large variability was reported in parents habit of checking on their children whilst asleep, in which only 18.95% (*n* = 29) checked three times or more per night, whilst infrequent parental checking of only once (*n* = 52, 33.99%), or twice (*n* = 46, 30.07%), and no parental checking (*n* = 26, 16.99%) were reported.

Results of the FAI questionnaire found that just under a quarter (n = 46, 23.1%) of the included participants reported a positive TMD status. Further logistic regression analysis (Table 5) revealed that children with social phobia were 2.6 times more likely to develop TMD disorder (*p* = *0.035*). Similarly, an increase in child’s age by 1 year increases the odds of developing TMD by 1.14 times (*p* = *0.037*). In addition, an association was also evident between those children showing signs of sleep bruxism and TMD, whereby those with bruxism are 5.5 times more likely to develop TMD (*p* = *0.012*).

**Table 5:** A binominal logistic regression model showing association of various factors on the odds of temporomandibular joint disorders (TMD)

	B	S.E	Wald	df	Sig	Odds ratio	95% Confidence interval for odds ratio	
							Lower	Upper
Age	0.131	0.063	4.355	1	0.037*	1.140	1.008	1.290
Nationality	–0.201	0.469	0.183	1	0.669	0.818	0.326	2.052
Relationship to the child	–0.149	0.437	0.117	1	0.733	0.861	0.366	2.028
History of COVID-19 infection of child or a family member	0.440	0.390	1.268	1	0.260	1.552	0.722	3.335
Overall anxiety disorder	–0.331	0.713	0.215	1	0.643	0.718	0.177	2.907
Panic/agoraphobia	0.544	0.489	1.238	1	0.266	1.722	0.661	4.487
Separation anxiety disorder	–0.026	0.516	0.002	1	0.960	0.975	0.355	2.680
Physical injury fears	0.186	0.498	0.139	1	0.709	1.204	0.454	3.194
Social phobia	0.972	0.460	4.454	1	0.035^*^	2.642	1.072	6.515
Obsessive compulsive disorder	–0.256	0.482	0.282	1	0.595	0.774	0.301	1.991
Generalised anxiety disorder	–0.161	0.491	0.107	1	.743	0.851	0.325	2.229
Sleep bruxism	1.709	0.679	6.327	1	0.012^*^	5.523	1.458	20.913
Constant	–2.945	0.798	13.610	1	< 0.001	0.053		

*B* co-efficient; *S.E* standard error; *Wald* Wald statistic value, *df* degree of freedom; Sig, *p* value; ^*^, statistically significant

Changes in children’s dietary habits and brushing practises are presented in Table 6. An increased consumption of food and sweets was reported in 51.8% and 62.8% of the participants, respectively. Furthermore, worsening of brushing habits was reported in approximately a third (31.2%) of the participants. A different pattern in terms of drinking more carbonated (fizzy/soft) drinks was noted, whereby 77.9% of participants indicated that their children did not drink more carbonated drinks during the COVID-19 pandemic-associated social restrictions. Of all the questions and eight factors included in the binomial logistic regression models, only age of the child was found to be statistically significant (*p* < 0.001) in relation to eating more sweets. An increase in age of the child was associated with decreased odds of eating more sweets during the COVID-19 social restriction period (odds ratio = 0.830, 95% confidence interval = 0.749—0.920) (Table 7).

**Table 6:** Change in dietary and brushing habits amongst participants

		Frequency (percentage)
Eating more food	Unfavourable change	103 (51.8%)
	Favourable change	96 (48.2%)
Eating more sweets	Unfavourable change	125 (62.8%)
	Favourable change	74 (37.2%)
Drinking more carbonated drinks	Unfavourable change	44 (22.1%)
	Favourable change	155 (77.9%)
Brushing habit worsened	Unfavourable change	62 (31.2%)
	Favourable change	137 (68.8%)

**Table 7:** A binomial logistic regression model showing association of various factors on the odds of excessive sweet consumption

	B	S.E	Wald	df	Sig	Odds ratio	95% Confidence interval for odds ratio	
							Lower	Upper
Age	– 0.186	0.052	12.650	1	< 0.001^*^	0.830	0.749	0.920
Overall anxiety disorder	– 0.073	0.631	0.013	1	0.908	0.930	0.270	3.201
Panic/agoraphobia	– 0.427	0.426	1.002	1	0.317	0.653	0.283	1.505
Separation anxiety disorder	– 0.150	0.437	0.118	1	0.731	0.861	0.366	2.026
Physical injury fears	0.383	0.435	0.777	1	0.378	1.467	0.626	3.440
Social phobia	0.190	0.399	0.226	1	0.635	1.209	0.553	2.642
Obsessive compulsive disorder	0.144	0.425	0.114	1	0.736	1.154	0.502	2.655
Generalised anxiety disorder	– 0.239	0.409	0.342	1	0.559	0.787	0.353	1.756
Constant	2.301	0.576	15.948	1	< 0.001	9.987		

*B* co-efficient; *S.E* standard error; *Wald* Wald statistic value, *df* degree of freedom; Sig, *p* value; ^*^statistically significant

## Discussion

This study is one of the first to explore the prevalence rates of anxiety, sleep bruxism, TMD and changes in dietary/brushing habits and its possible association with the social restrictions imposed by the COVID-19 pandemic within a cohort of children and adolescents. The main finding of this study is the presence of elevated levels of overall anxiety in approximately one-third of the included participants, with no previous history of such concerns prior to the pandemic. This finding is consistent with other studies showing increased anxiety symptoms in children and adolescents during the current pandemic (Khan et al. 2021; Kostev et al. 2021; Śniadach et al. 2021). In addition, elevated levels of overall anxiety were shown to be more prevalent in younger (≤ 9-year old) compared to older (≥ 10-year old) aged children. In terms of specific anxiety domains, this study reports a high prevalence of social phobia, which is in keeping with established literature (Cartwright-Hatton et al. 2006; Franz et al. 2013). We surmise that this could indicate the fear of the pandemic itself due to the associated social restrictions, and the fear of the risk of getting infected.

Results of this study report sleep bruxism in 5.7% of the children and adolescents which is towards the lower end of those reported in previous literature (3.5% to 40.6%) (Manfredini et al. 2013). This wide range of prevalence rate is possibly related to the differences in the age range of included participants, study design and instrument used. The variability of parental access to their children during their sleeping hours is also difficult to standardise resulting in an unintentionally introduced bias. In the current study, access of parents to their children during sleep time was limited by a number of factors, such as infrequent parental checking, which could have underestimated the prevalence rates of reported sleep bruxism in this study. Therefore, this specific result should be interpreted with caution since no pre-pandemic baseline sleep bruxism prevalence rates amongst the Qatari population are available. Nevertheless, this data can act as a comparator for future studies.

In addition, this study reported symptoms of TMD in almost a quarter of participating children and adolescents, who otherwise suffered no previous history of TMD. In similarity to sleep bruxism, a wide variability of TMD prevalence ranging between 7.3 and 30.4% is reported in the literature (Christidis et al. 2019). Such variability could be related to the multiple assessment tools used and the lack of standardised examination protocols. The prevalence rates of TMD, shown in this study, are towards the higher end of the reported prevalence range in the literature. Although the lack of local pre-pandemic TMD data precludes linking such higher prevalence to the COVID-19 pandemic, TMD in this study was found to be associated with increased social phobia, therefore indicating a possible relationship with the COVID-19 pandemic social restrictions. Of interest, in a recent study involving adults participants, an increase in psychosocial distress was linked to an increase in bruxism and TMD symptoms during the COVID-19 pandemic (Colonna et al. 2021). Although the FAI is a validated self-rated questionnaire, used for screening children/adolescents with TMD (Rigoldi Bonjardim et al. 2008; de Santis et al. 2014), some concerns have been raised in relation to its accuracy when used in different languages, and with young children (de Santis et al. 2014). To overcome such limitations, the questions were read and explained to the parents during a face-to-face or telephone interview by dental specialists.

Furthermore, this study highlighted worrying changes in children’s and adolescents’ dietary and brushing habits over the course of the recent COVID-19 pandemic, which is in line with the negative impact of COVID-19 on the dietary habits reported globally (Campagnaro et al. 2020; Bennett et al. 2021). An increase consumption of food, specifically sweets, was apparent in a large proportion of participants. A higher consumption of sweets and the reduction in tooth brushing habits in the younger children were evident. Such changes, specifically if combined, could most likely result in an increased risk of dental caries and periodontal disease which warrants better communication between dental health services during any pandemic-related restrictions.

This study sets out one of the first reports in the Middle East and North Africa (MENA) region of how COVID-19 has impacted children’s and adolescents’ oral health when analysed in the context of higher anxiety and social phobia due to the social restrictions. This is a critical finding that should inform public health policy and health care delivery during the current late stage of the pandemic and future possible pandemics. Measures aimed at early identification and management of anxiety and oral health in children within the specific context of pandemics need to be further developed. Qatar was reasonably successful in delivering virtual mental health care through telephone and video consultations; however, there is little data to suggest whether these measures were ever designed for children and whether children benefited (Karim et al. 2020). Qatar also developed an online dental patient advice website created to help patients identify and possibly manage the most common dental emergencies with a helpline for those requiring further assistance. However, the abovementioned website did not include advice on identification and seeking help for sleep bruxism, TMD or change in diet/brushing habits and did not link social restrictions with psychological anxiety and its impact on oral health.

Results of this study further aim to inform a better designed and accessible information campaign. There is merit in utilising information technology and virtual dentistry clinics that are not limited by the quarantine restrictions for early identification of dental problems associated with psychological distress in children (Ali et al. 2022). In addition, the utilisation of mHealth (in means of text messages and phone applications) is regarded as a promising adjunct clinical tool for spreading awareness and health education aiming at preventing and promoting oral health during the pandemic (Luzzi et al. 2021). Although currently, most countries have lifted the COVID-19 restriction-related measures, such as social distancing, the pandemic continues to infect people in new waves of infection as new variants of the coronavirus evolve. It follows that health systems should support health action plans that recognise the, yet to be fully understood impact, of this waxing and waning pandemic and focus on general advice, early identification and measures supporting mental health and oral health in the young generation.

This study was subjected to some unavoidable limitations due to the nature of the pandemic and its subsequent restrictions to patient recruitment and examination. The recruitment of patients followed a convenient sampling technique of those attending two specific dental centres within the State of Qatar which might have affected the generalisability of the results. Such design was performed due to the applied lockdown restrictions and inability to conduct the study in a wider random setting. In addition, data collected relied on parental questioners and specific assessments tools which, whilst possessing advantages, have inherent limitations such as their overall reliability and sensitivity that are acknowledged by the authors. In addition, in this study, the prevalence of sleep bruxism and TMD relied solely on history taking; however, adjunct clinical examination would have been beneficial to further affirm these results. Unfortunately, due to the pandemic restrictions, clinical examination was not performed due to restrictions applied in the dental office. Although well-designed large multi-centre clinical studies are required to ascertain the impact of pandemic restriction-related measures on the mental and dental health of children and adolescents, such studies are not easily planned and executed due to the level of restriction of such unprecedented global pandemic.

## Conclusion

Within the limitations of this study, the results highlight an increase in anxiety disorders pertaining mainly to social phobia was evident during pandemic-related social restrictions, which could result in unwanted oral consequences such as TMD. Negative changes in children’s and adolescents’ dietary and brushing habits were also evident.
